# The role of amputative and non-amputative foot deformities severity in the risk for diabetic ulceration classification systems building: a cross-sectional and case-control pilot investigation

**DOI:** 10.11604/pamj.2019.33.103.17684

**Published:** 2019-06-11

**Authors:** Aristomenis Kossioris, Nicholas Tentolouris, Chariclia V Loupa, Minos Tyllianakis

**Affiliations:** 1 Department of Neurology, General Hospital of Athens “G. Gennimatas”; School of Medicine, University of Patras, Rio, Greece; 2 First Department of Propaedeutic and Internal Medicine, National and Kapodistrian University of Athens, “Laiko” General Hospital, Athens, Greece; 3 “Demetrios Voyatzoglou” Diabetic Foot Clinic, “A. Fleming” General Hospital, Athens, Greece; 4 Department of Orthopaedics, School of Medicine, University of Patras, Rio, Greece

**Keywords:** Diabetic foot ulceration, amputative foot deformities, non-amputative foot deformities, severity, ROC curve analysis

## Abstract

**Introduction:**

Foot deformities and amputations are parameters that have been studied as risk factors for diabetic foot ulceration (DFU). However, inclusion of “foot deformities” and “amputations” in a single, broad variable and with reference to the severity of these deformities, may better characterize subjects who are prone to develop DFU.

**Methods:**

The objective of the study was the examination of amputative and non-amputative foot deformities severity as risk factor for DFU in relation with the other established risk factors. A cross-sectional and case-control study was conducted from October 2005 to November 2016. One hundred and thirty-four subjects with type 1 and 2 diabetes, with and without active foot ulcers, participated. A structured quantitative interview guide was used. Univariate logistic regression analysis for the literature's established risk factors was performed, as well as for two versions of the “amputative and non-amputative foot deformities severity” variable. Subsequently, multivariate logistic regression analysis (MLRA) for three models and receiver operating characteristic (ROC) curve analysis were carried out.

**Results:**

From the MLRA, only PAD (peripheral arterial disease) was significant (OR 3.56, 95% CI 1.17-10.82, P=0.025 and OR 3.33, 95% CI 1.02-10.08, P=0.033). Concerning the ROC curve analysis of the models, the one with the three categories amputative and non-amputative foot deformities severity variable, had the greatest area under the ROC curve (0.763, P<0.001).

**Conclusion:**

A united variable for lower extremity amputations and other foot deformities with reference to their severity, could be more helpful to the clinicians in identifying patients with diabetes at risk for foot ulceration.

## Introduction

The development of ulcers, of catalytic etiology either intrinsic (e.g resulting from high plantar pressures due to prominent metatarsal heads) or extrinsic (e.g. resulting from a pebble during walking shoeless) [1], at feet of persons with diabetes (diabetic foot ulceration, DFU), can bring about serious complications both individually (amputation-related disability and increased mortality) and socially (economic burden of the health systems) [2]. According to the epidemiologic studies, the DFU risk factors that predominantly have been identified include peripheral neuropathy, peripheral arterial disease (PAD), structural foot deformities (hammer toes, claw toes, etc) and the history of amputation and/or previous ulceration [1, 2]. DFU is preventable applying appropriate interventions, therefore, various, but slightly different, risk classification systems with predictive value have been developed [2-4].

In literature [2, 4-13], foot structural deformities have been studied as risk factor for:

- First ulcer/s [6, 7]
- First ulcer(s) and recurrent ulcer(s) [8, 9] and
- Recurrent ulcer(s) [10, 13].

The foot deformities *per se*, have been administered by the researchers either as two separate entity groups:

- Amputations (amputative deformities) and/or
- Structural or foot deformities (non-amputative deformities) [6-10] or as a single entity group [13]:
- Structural or foot deformities (amputative and non-amputative deformities together).

The terms amputative and non-amputative, concerning the separation of amputations from the rest foot deformities in people with diabetes, are more accurate in relation to a potential use of the terms “extrinsic” for amputations and “intrinsic” for deformities such as claw toes or prominent metatarsal heads. A non-amputative deformity could have a cause outside of diabetic neuropathy, which is an intrinsic factor (e.g. hammer toes can be a result of trauma or inappropriate shoes) [14]. Foot deformities and their severity are parameters that have been studied in the past as risk factors for ulceration development in patients with diabetes [6, 9]. Although, the terms foot deformities and amputations are confusing in the literature with glaring example the recent IWGDF definitions and risk classification system of 201^5^ [5], in which amputations once is included in the term foot deformity (IWGDF definitions, p. 17), while another time is not (Table 1, p. 18). Since amputations are also deformities, the administration of foot deformities as a broad variable, including both amputative and non-amputative ones, is more precise. Severity of foot deformities only recently has been studied, precisely and with breadth, as a united variable including both amputative and non-amputative ones [13]. No study yet has examined the amputative and non-amputative foot deformities severity as risk factor for DFU in association with the established risk factors (peripheral neuropathy, PAD, history of previous ulceration).

**Table 1:** Frequencies of patients with diabetic foot disease characteristics

Characteristics	N	Results*
Sociodemographic		
Sex	(134)	Men= 67.9%; Women= 32.1%
Age (years)	(129)	64.9 ± 12.2
Companionship status	(75)	Living with others= 92.0%; Lonely living= 8.0%
Education level	(71)	Secondary= 47.9%; Tertiary= 35.2%; Primary= 16.9%
Labor market status	(94)	Outside= 80.9%; Inside= 19.1%
**Clinical**		
Diabetes type	(130)	Type 2= 92.3%; Type 1= 7.7%
Diabetic peripheral neuropathy	(114)	57.9%
PAD**	(110)	40.0%
Amputative foot deformities^†^	(105)	20.0%
Non-amputative foot deformities (Pes planus, hallux valgus, hammer toes, etc.)^§^	(106)	43.4%
Severity of amputative and non-amputative foot deformities (four categories)	(98)	None= 36.7%; Mild= 34.7%; Moderate= 26.5%; Severe= 2.0%
Severity of amputative and non-amputative foot deformities (two categories)	(98)	None/Mild= 71.4%; Moderate/Severe= 28.6%
Active foot ulceration	(134)	44.8%
History of previous foot ulceration	(103)	37.9%
Prevalence of appropriate footwear	(92)	53.3%

* Results are % or median (interquartile range) or mean ± SD

** Peripheral arterial disease

† Amputative foot deformities frequencies: Hallux or ray amputation=10.7%; Lesser toe amputation=9.6%

§ Non-amputative foot deformities frequencies: Hammer toes=14.6%; Claw toes=13.6%; Hallux valgus=12.6%; Prominent metatarsal heads=12.5%; Pes planus=7.8%; Charcot neuroarthropathy=1.8

The aim of this study was the examination of amputative and non-amputative foot deformities severity as risk factor for DFU in relation with the other established risk factors, as well as of the participants' sociodemographic and clinical characteristics.

## Methods

### Study design

The study was a cross-sectional, case-control research.

### Setting

The research came about at three diabetic foot clinics of general hospitals and one wound unit of a special hospital in a large capital city. Ethics approvals were granted by the hospitals' scientific committees.

### Subjects

The study participants were individuals with type 1 and 2 diabetes and with or without foot ulcers. Patients with cognitive disturbances were excluded from the study.

### Recruitment

One hundred and thirty-four patients were conveniently approached by the head investigators during their scheduled first or subsequent visit to the healthcare facilities, from October 2005 to November 201^6^. The sample size was calculated implementing approximately the Garson's [15] rule of thumb whereby the number of cases in the smaller of the two binary outcomes in binary logistic regression divided by the number of predictor variables should be at least 20 [15]. All participants were enrolled after providing written informed consent.

### Data collection

For the collection of the data, a structured quantitative interview guide with closed-ended questions was used. The principal researchers interviewed one-on-one each patient gathering and recording demographic and clinical data.

### Measurements

The parameters that were measured were related to:

- Sociodemographic characteristics: sex, age (years), companionship status, education level, labor market status and
- Clinical characteristics: Diabetes type, diabetes duration (years), treatment type, HbA1c (%), blood glucose level (mg/dL), presence or absence of retinopathy, renal complications, hypertension, coronary artery disease, diabetic peripheral neuropathy (somatic sensorimotor), PAD, amputative foot deformities, non-amputative foot deformities, severity of amputative and non-amputative foot deformities, history of previous foot ulceration, active foot ulceration, risk classification for foot ulceration development and appropriate footwear prevalence.

### Instrumentation - procedures

For the measurement of sociodemographic characteristics, appropriate interview guide items were utilized. The items asked primarily objective information, thus, the interview guide was subjected only to validity investigation. All the interview guide items were tested by applying the face validity method. Regarding the clinical characteristics of diabetes type, diabetes duration, treatment type, HbA1c (%), blood glucose level, presence or absence of retinopathy, renal complications, hypertension, coronary artery disease, active foot ulceration and history of previous foot ulcer(s), initially, appropriate interview guide items were used, and afterwards the researchers confirmed the data's validity by checking the biochemical and hemodynamic tests, as well as the other medical files of the participants. Diabetic foot ulcer was defined as full thickness break of the skin, at least of Wagner stage 1 [16], infected or not and developed distal to the malleoli. As for the loss of protective sensation attributable to peripheral neuropathy, it was diagnosed by applying the 10g monofilament and the vibration perception threshold test [1]. In terms of PAD, the diagnosis was based on duplex ultrasonography with >50% vessel stenosis being indicative [17, 18]. Concerning the foot deformities (both amputative and non-amputative), they were diagnosed by the physicians of the research team by utilizing inspection where needed (e.g. for diagnosing Charcot's neuroarthropathy) by checking previous imaging examinations [1]. Respecting the classification of the amputative and non-amputative foot deformities severity, that was founded in the Waaijman *et al*. [13, 1^9^] guidelines. As for the risk for DFU classification system, the risk classification based on the comprehensive foot examination of Boulton *et al*. [1, 4] was used. With reference to the prevalence of appropriate footwear, the shoes or aids that were accompanied by literature evidence (comprising expert opinion) concerning effectiveness (conventional off-the-self, semi-customized and customized diabetic shoes or slippers-sandals, running shoes, half-shoes, total contact casts and removable walkers) were counted [20, 21].

### Data analysis

At first, because there were only two observations for the severe category of the Waaijman *et al*. [13, 19] variable from the small pilot sample and of the fact that the recommended smallest of the classes of the depended variable in a regression model is at least 10 events per parameter [15], the amputative and non-amputative foot deformities severity parameter from a four categories variable (none, mild, moderate, severe), yielding high logistic coefficients [15], was altered to a three classes one (none, mild and moderate/severe) with the last two categories combined and following this to a two classes one (none/mild and moderate/severe) with the first and last two categories combined.

### Statistical analysis

Descriptive and inferential statistical analysis took place while utilizing the IBM SPSS 24 software package. Within the bounds of descriptive analysis, the frequencies of the sociodemographic and clinical characteristics were estimated. With respect to the inferential statistical analysis, univariate logistic regression analysis for the risk classification system of Boulton *et al*. [1, 4] risk factors was performed, as well as for both the three and two categories versions of the amputative and non-amputative foot deformities severity variable. Subsequently, multivariate logistic regression analysis was carried out for examining three regression models:

- The first (model 1) with the risk classification system of Boulton *et al*. [1, 4] risk factors
- The second (model 2) with the above factors, but with a replacement of foot deformities and amputations variables with the three categories version of the amputative and non-amputative foot deformities severity variable and
- The third (model 3) with the same factors, but with the two categories version of the amputative and non-amputative foot deformities severity variable instead of the three categories one.

For the multivariate regression analyses, the “enter”variable selection method was used and 5% probability criterion was set for the variables to enter the models. After the multivariate regression investigation of the aforesaid variables, and considering that the research purpose was prediction [15], a ROC (receiver operating characteristic) curve analysis for compering the yielded models took place.

## Results

### Descriptive

With regards to the sociodemographic characteristics, 67.9% of the participants were men, with the total sample's mean age being 64.9±12.2. Ninety-two per cent were living with others, 47.9% had just primary and secondary education and the 80.9% were outside of the labor market. As for the clinical characteristics, 92.3% of the study subjects had type 2 diabetes, 57.9% peripheral neuropathy, 40.0% PAD, 43.4% non-amputative foot deformities, while 20.0% amputative foot deformities, 53.3% wore appropriate footwear and of the controls, 51.4% were at no risk for DFU. All the descriptive results are shown in detail in Table 1.

### Inferential

The univariate logistic regression analysis, in terms of the variables that were involved in the three models (1, 2 and 3) was significant (P≤0.05) for diabetic peripheral neuropathy (OR 3.80, 95% CI 1.66-8.70, P=0.002), PAD (OR 4.14, 95% CI 1.84-9.32, P=0.001), amputative foot deformities (OR 2.78, 95% CI 1.04-7.45, P=0.042), history of previous foot ulceration (OR 3.79, 95% CI 1.64-8.77, P=0.002) and moderate/severe foot deformities from the two categories amputative and non-amputative foot deformities severity variable (reference category: none/mild) (OR 2.78, 95% CI 1.13-6.86, P=0.026) (Table 2, Table 3, Table 4).

**Table 2:** Univariate logistic regression analysis for the comprehensive foot examination classification system DFU* risk factors with two foot deformities-related variables (the multivariate logistic regression analysis did not yield statistically significant results); model 1

	Univariate analysis	
Variable**	OR (95% CI)	*P*
Diabetic peripheral neuropathy	3.80 (1.66-8.70)	0.002
PAD^†^	4.14 (1.84-9.32)	0.001
Non-amputative foot deformities	1.08 (0.50-2.34)	0.852
Amputative foot deformities	2.78 (1.04-7.45)	0.042
History of previous foot ulceration	3.79 (1.64-8.77)	0.002

* Diabetic foot ulceration

** The variables have been ordered according to the comprehensive foot examination classification system

† Peripheral arterial disease

**Table 3:** Univariate and multivariate logistic regression analysis for the comprehensive foot examination classification system DFU risk factors with a single foot deformities-related variable and three categories of severity (none, mild and moderate/severe); model 2

	Univariate analysis		Multivariate analysis	
Variable	OR (95% CI)	*P*	OR (95% CI)	*P*
Diabetic peripheral neuropathy	3.80 (1.66-8.70)	0.002		
PAD*	4.14 (1.84-9.32)	0.001	3.33 (1.10-10.08)	0.033
Amputative and non-amputative foot deformities severity				
None	Reference category			
Mild	0.97 (0.36-2.57)	0.943		
Moderate/severe	2.73 (0.99-7.57)	0.053		
History of previous foot ulceration	3.79 (1.64-8.77)	0.002		

* Peripheral arterial disease

**Table 4:** Univariate and multivariate logistic regression analysis for the comprehensive foot examination classification system DFU risk factors with a single foot deformities-related variable and two categories of severity (none/mild and moderate/severe); model 3

	Univariate analysis		Multivariate analysis	
Variable	OR (95% CI)	*P*	OR (95% CI)	*P*
Diabetic peripheral neuropathy	3.80 (1.66-8.70)	0.002		
PAD*	4.14 (1.84-9.32)	0.001	3.19 (1.07-9.52)	0.037
Amputative and non-amputative foot deformities severity				
None/Mild	Reference category			
Moderate/Severe	2.78 (1.13-6.86)	0.026		
History of previous foot ulceration	3.79 (1.64-8.77)	0.002		

* Peripheral arterial disease

**Figure 1:**
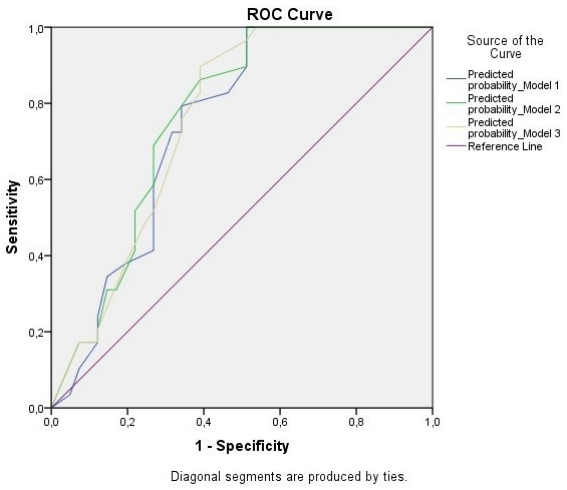
ROC curve analysis for the three predicting models

In regards to the model 1 multivariate logistic regression analysis (MLRA), none significant variable was yielded (Table 2). As for the model 2 and model 3 MLRA, only PAD was significant (OR 3.56, 95% CI 1.17-10.82, P=0.025 and OR 3.33, 95% CI 1.10-10.08, P=0.033 respectively) (Table 3, Table 4). Concerning the ROC curve analysis of the three models, model 2 had the greatest area under the ROC curve (0.763, P<0.001) (Figure 1, Table 5).

**Table 5:** Areas under the ROC curves

	Area under the curve	95% CI	*P* value
Model 1	0.741	(0.63-0.86)	0.001
Model 2	0.763	(0.65-0.87)	<0.001
Model 3	0.754	(0.64-0.87)	<0.001

## Discussion

Even though this was a small-sized pilot study, given the fact that coping with a problem as common as diabetic foot ulceration necessitates a larger cohort, for testing the feasibility of the methodology that was chosen [22], it managed to bring in useful results.

The most important finding of the study was the fact that both models 2 and 3, with the three and two categories amputative and non-amputative foot deformities severity variable, by the ROC curve analysis were shown to have greater areas under the ROC curve (0.763, P<0.001 and 0.754, P<0.001 respectively) than the Boulton *et al*. -based model's area (model 1) [4] with model 2 showing the greatest difference (0.022) demonstrating the optimal classification, and hence predictive, ability [15].

The second most weighty detection of the research was the designation of PAD as a DFU risk factor by the MLRA of both 2 and 3 models variables (OR 3.56, 95% CI 1.17-10.82, P=0.025 and OR 3.33, 95% CI 1.10-10.08, P=0.033 respectively). PAD has been identified as a major risk factor for DFU at patients with diabetes feet by several pivotal studies [9, 10, 23, 24].

By the univariate logistic regression analysis, the parameters of peripheral neuropathy, PAD, amputative foot deformities and history of previous ulceration, in concordance with the literature [8, 9, 23-26] were discovered to be significantly associated with the presence of active foot ulceration.

In terms of the sociodemographic and the clinical characteristics that were not examined in the context of inferential analysis, by the descriptive analysis, the prevalence of wearing appropriate footwear (53.3%) was in consonance with the literature [27-30], in which the prevalence in question was calculated to be 52% [21].

## Conclusion

A single, united variable for lower extremity amputations and other foot deformities with reference to their severity and with ≥2 severity classes, could be more helpful to the clinicians in identifying patients with diabetes at risk for foot ulceration.

New, improved classification or stratification systems for predicting intents, replacing established ones, are emerging constantly in the literature [31]. Therefore, we encourage the diabetic foot-related scientific associations to consider the possibility of modifying the current risk for DFU classification systems according to the findings of the present investigation or future, more powered, relevant studies.

### What is known about this topic

- Lower extremity amputations and foot structural deformities such as hammer and claw toes, along with somatic sensorimotor peripheral neuropathy, PAD and the history of previous ulceration constitute the literature's established risk factors for DFU;
- DFU is preventable applying appropriate interventions and therefore various but slightly different risk classification systems for medical check-up or screening, based on the five established risk factors, have been developed.

### What this study adds

- Inclusion of amputations (amputative deformities) and foot deformities such as prominent metatarsal heads and hammer or claw toes (non-amputative deformities) in a single, broad variable with reference to their severity characterizes better the persons who are prone to develop DFU;
- A single, united variable for amputative and non-amputative foot deformities with reference to their severity and with ≥2 severity classes, together with the other DFU established risk factors produce risk classification systems of better predictive ability.

## Competing interests

The authors declare no competing interests.
